# Catching allergy by a simple questionnaire

**DOI:** 10.1186/s40413-015-0067-y

**Published:** 2015-06-11

**Authors:** Maurizio Galimberti, Giovanni Passalacqua, Cristoforo Incorvaia, Vincenzo Castella, Maria Teresa Costantino, Beniamino Cucchi, Sebastiano Gangemi, Gilda Nardi, Paolo Raviolo, Paola Rottoli, Nicola Scichilone, Nico Sciolla, Germano Bettoncelli, Massimo Landi, Erminia Ridolo, Serena Buttafava, Paola Puccinelli, Giorgio Walter Canonica, Alessandro Fiocchi, Franco Frati

**Affiliations:** Allergology Department, Ospedale Maggiore della Carità, Novara, Italy; Allergy and Respiratory Diseases, University Department of Internal Medicine, Genoa, Italy; Allergy/Pulmonary Rehabilitation Unit, ICP Hospital, Milan, Italy; Pediatric Unit, Riuniti Hospital, Tortona, Italy; Allergy Unit, Carlo Poma Hospital, Mantova, Italy; Allergy Unit, Riuniti Hospital, Rivoli, Italy; Allergy and Clinical Immunology Unit, G. Martino Hospital, Messina, Italy; Immunohaematology and Transfusion Service, ASUR Zona 13, Ascoli Piceno, Italy; Department of Clinical Medicine and Immunological Sciences, Santa Maria alle Scotte Hospital, Siena, Italy; Allergy Service/Pneumology Unit, Cervello Hospital, Palermo, Italy; National Healthcare System, Brescia, Italy; National Healthcare System, ASL TO3, Turin, Italy; Department of Clinical and Experimental Medicine, University of Parma, Parma, Italy; Pediatric Hospital Bambino Gesù, Rome, Italy; Medical, Scientific and Regulatory Department, Stallergenes Italy, Via Tibullo 2, 20151, Milan, Italy

**Keywords:** Allergy, Allergic rhinitis, Allergy testing, Primary care physicians, Pharmacists

## Abstract

**Background:**

Identifying allergic rhinitis requires allergy testing, but the first-line referral for rhinitis are usually primary care physicians (PCP), who are not familiar with such tests. The availability of easy and simple tests to be used by PCP to suggest allergy should be very useful.

**Methods:**

The Respiratory Allergy Prediction (RAP) test, based on 9 questions and previously validated by a panel of experts, was evaluated in this study.

**Results:**

An overall number of 401 patients (48.6% males, age range 14–62 years) with respiratory symptoms was included. Of them, 89 (22.2%) showed negative results to SPT, while 312 (77.8%) had at least one positive result to SPT. Cohen’s kappa coefficient showed that all questions had an almost perfect excellent agreement between pre and post-test. The algorithm of decision-tree growth Chi-squared Automatic Interaction Detector showed that answering yes to the question 4 (Your nasal/ocular complains do usually start or worsen during the spring?), 6 (Did you ever had cough or shortness of breath, even during exercise?) and 8 (Do you use nasal sprays frequently?) gave a probability to have a positive SPT of 85%.

**Conclusions:**

These findings show that RAP test can be proposed as an useful tool to be used by physician other than allergists when evaluating patients with rhinitis, suggesting the need of allergy testing.

**Electronic supplementary material:**

The online version of this article (doi:10.1186/s40413-015-0067-y) contains supplementary material, which is available to authorized users.

## Background

Allergic rhinitis (AR) is the most frequent immune-mediated disorder, and its prevalence is still increasing worldwide, as recently underlined in the Gene Environment Interactions in Respiratory Diseases (GEIRD) epidemiological study [1]. AR is defined as a symptomatic disorder of the nasal mucosa, due to an IgE-mediated reaction that follows the immediate contact with an offending allergen. The major symptoms of AR are nasal itching, sneezing, rhinorrhea and obstruction, that are spontaneously reversible or may be controlled by adequate treatment [2]. AR may also be part of the Severe Chronic Upper Airways Disease (SCUAD) [3] and is frequently associated with comorbidities such as rhinosinusitis, asthma, conjunctivitis, nasal polyposis, and sleep disturbances, which make the diagnostic approach more complex [4]. Nevertheless, AR is still often considered as a “trivial” disease, that can be easily manageable by the patient himself or by healthcare providers other than physicians. Instead, its health and social impact is substantial, considering the costs, either direct (expenditure for drugs, access to medical care) or indirect (absenteeism, presenteism, decreased school/work performance) [5-7]. Finally, AR is frequently associated with asthma but, more importantly, it represents the more relevant independent risk factor for asthma onset [8]. In this complex context, primary care physicians and also pharmacists are usually the first-line referral for AR patients, but thus far there is no easy, simple and self-administered test to be used in the primary care physician setting to discriminate a suspicion of AR, so that the patient can be subsequently referred to the allergist for a more detailed or confirmatory diagnosis by skin prick test (SPT), *in vitro* IgE assay, specific provocation test or molecular-based diagnosis.

The aim of this study was to set-up a simple clinical questionnaire, to be used in primary care, able to identify with a good positive predictive value the presence of AR.

## Methods

An initial version of the questionnaire, named Respiratory Allergy Prediction (RAP) test, involving 10 yes/no questions was agreed by a panel of experts, based on literature data, personal experience and clinical observations. This first version was then used in a pilot study including 40 patients (approved by the Ethic Committee of the Ospedale Maggiore della Carità, Novara, Italy, N. CE 45/08) [9]. The questionnaire underwent a pre- and re-test procedure, followed by the standard diagnostic work-up for allergy diagnosis. The preliminary evaluation allowed to obtain a final version of the RAP questionnaire in 9 questions, as reported in Table 1, that was evaluated and validated in a large sample of patients, object of the present observational, multicenter, prospective study. The results of the RAP test (including test-retest analysis) were compared to the results of SPTs, obtained in a blinded fashion.

**Table 1:** **The 9-question RAP questionnaire**

1	Do you have parents/relatives suffering from rhinitis and/or asthma?
2	Do you suffer from itchy/red/watery eyes during the year?
3	Do you experience runny nose/nasal obstruction/nasal itching for many consecutive days?
4	Your nasal/ocular complaints do usually start or worsen during the spring?
5	Have you ever heard wheezing breath?
6	Did you ever had cough or shortness of breath, even during exercise?
7	Do you have nocturnal awakenings due to shortness of breath or cough?
8	Do you use nasal sprays frequently?
9	Do you feel that your nasal symptoms worsen in dusty environments?

Y/N answers allowed.

The primary objective was to set-up a simple clinical questionnaire in Italian language for the primary care physicians able to suggest AR with a good positive predictive value.

The primary efficacy variables were:

- Validity, the capacity of the questionnaire to recognize AR compared with an evaluation by SPT, performed in blinded fashion by the physician during the visit;
- Reliability. The reliability of the questionnaire, i.e. the capacity to provide stable measurements in the case of the stability of environment, was assessed by repeating the administration of the questionnaire on all included patients.

The secondary objectives were the evaluation of the predictivity of each question concerning respiratory allergy and characterization of allergic condition in the studied sample.

All the assessments in the study were in blinded condition and the data were registered in case report form.

### Patients

The patients were enrolled consecutively among those referred for the first time because of a suspected AR, as suggested by symptoms such as rhinorrhea, itching, sneezing, and nasal obstruction, with or without lower respiratory symptoms, to 9 Allergy Units distributed over the Italian territory. The Units were: Allergy and Clinical Immunology Unit, Ospedale Maggiore della Carità, Novara; Pediatric Unit, Riuniti Hospital, Tortona; Allergy Unit, Riuniti Hospital, Rivoli; Pediatric Unit, Riuniti Hospital, Rivoli; Allergy Unit, Carlo Poma Hospital, Mantova; Allergy and Clinical Immunology Unit, G. Martino Hospital, Messina; Immunohaematology and Transfusion Service, Azienda Sanitaria Unica Regionale Zona 13, Ascoli Piceno; Department of Clinical Medicine and Immunological Sciences, Santa Maria alle Scotte Hospital, Siena; Allergy Service/Pneumology Unit, Cervello Hospital, Palermo.

Inclusion criteria were: patients reporting respiratory symptoms; age between 6 and 60 years; written informed consent to participate to the study; patients already eligible for SPT.

Exclusion criteria were: a previous physician-based diagnosis of AR and/or stably treated with symptomatic medications (e.g. antihistamines, nasal corticosteroids, nasal decongestants) or allergen immunotherapy, occurrence of skin disorders such as atopic dermatitis, dermographism or other pathologies potentially interfering with skin reactivity; patients with psychiatric disorders.

The patients had to fill the RAP questionnaire two times (test-retest) before undergoing the diagnostic procedures by SPTs.

Table 1 shows the 9 items of the questionnaire.

SPTs were performed in all centres with the same panel of commercial extracts (Stallergenes Italy, Milan), including dust mites, grasses, Parietaria, birch, hazelnut, olive, cypress, mugwort, ragweed, cat/dog epithelia, and cockroach, that represent the most common sensitizers in Italy. Positive (histamine) and negative (diluent) controls were also used, and the results were read after 20 minutes according to European guidelines [10].

### Ethical aspects

Written informed consent was obtained by each participant before entering the study, and in the case of minors was obtained from next of kin, caregivers, or guardians. The study was conducted in accordance with good clinical practice guidelines. The ethics committees at each of the participating sites approved the study.

### Statistical analysis

A heuristic sample size of about 450 patients was hypothesized as sufficient to evaluate the efficacy of the questionnaire. To interpret the efficiency of the questionnaire in predicting allergy, we used the algorithm of decision-tree growth Chi-squared Automatic Interaction Detector (CHAID), a method that automatically searches for important patterns and relationships and quickly uncovers hidden structure even in highly complex data. In other words, subdivisions of the tree are constructed so as to maximize the differences in response between the two groups of the split [11].

The variable “target” used is the SPT, classified in two categories of positivity: YES and NO. The explanatory variables are represented instead by a questionnaire with 9 questions administered to patients during the examination. The reliability of agreement between pre and post-test were analyzed by Cohen’s kappa coefficient. The measure calculates the degree of agreement in classification over that which would be expected by chance. Landis and Koch [12] gave the following values for interpreting k values:

- 0.01 to 0.20, slight agreement;
- 0.21 to 0.40, fair agreement;
- 0.41 to 0.60, moderate agreement;
- 0.61 to 0.80, substantial agreement and almost perfect from
- 0.81 to 1 almost perfect agreement.

Data were also plotted in Receiving Operating Curve (ROC), in which area under the curve (AUC) corresponds to a likelihood that a positive response is more frequent than a negative response. An ideal model provides an AUC as 1; a random model, in which a positive response to the presence of a symptom is not correlated with the positivity to SPT, provides an AUC as 0.5.

## Results

A total of 426 patients were enrolled and included in the database. Of them, 25 patients erroneously received a version of the questionnaire and were therefore excluded, reducing to 401 (48.6% males, mean age 26.1 years, age range 14–62 years) the number of patients analyzed.

Patients were referred to the specialists by primary care physicians in 292/401 (72.8%), pediatricians in 39/401 (9.7%), Ear Nose Throat (ENT) specialists in 28/401 (7%), pulmonologists in 14/401 (3.5%) and ophtalmologists in 6/401 (1.5%).

Their main demographic and clinical characteristics are reported in Table 2. The sample was mainly composed by non-smokers (297/401, 74.5%). Table 3 shows the differences according to smoking habit, that were not statistically significant.

**Table 2:** **Main demographic and clinical characteristics of the 401 patients**

	**N**	**%**
**Male/female**	195/206	48.6/51.4
**Mean age (range)**	26.1 (14–62)	
**Smoking habit**		
Current	56	14
Never/former	343	86
Allergic rhinitis yes/no	365/36	91/9
**Allergic rhinitis classification**		
Mild intermittent	107	29.3
Mild persistent	80	21.9
Moderate/severe intermittent	55	15.1
Moderate/severe persistent	123	33.7
Asthma yes/no	143/258	35.7/64.3
**Asthma classification**		
Intermittent	84	58.7
Mild persistent	34	23.8
Moderate persistent	24	16.8
Severe persistent	1	0.7
**Conjunctivitis yes/no**	238/163	59.4/40.6

**Table 3:** **Differences in severity of allergic rhinitis according to smoking habit**

**Observed frequencies**				**Significance by Fisher’s exact test**		
**Severity of AR/smoking habit**			**Total**			
	**Current**	**Never/former**			**Current**	**Never/former**
Mild intermittent	10	96	96	Mild intermittent	<	>
Mild persistent	17	63	80	Mild persistent	>	<
Moderate/severe intermittent	11	43	43	Moderate/severe intermittent	>	<
Moderate/severe persistent	17	106	106	Moderate/severe persistent	<	>
Total	56	343	399			
**Theoretical frequencies**				**Percentage/Column**		
**Severity of AR/smoking habit**			**Total**			
	**Current**	**Never/former**			**Current**	**Never/former**
Mild intermittent	14.877	91.123	106.000	Mild intermittent	17.857	27.988
Mild persistent	11.228	68.772	80.000	Mild persistent	30.357	18.367
Moderate/severe intermittent	7.579	46.421	54.000	Moderate/severe intermittent	19.643	12.536
Moderate/severe persistent	17	106	123.000	Moderate/severe persistent	30.357	30.394
Total	56	343	399	Total	100	100

The most commonly reported symptoms were nasal symptoms in 368/401 (91.8%), followed by ocular symptoms in 238/401 (59.4%) and bronchial symptoms in 205/401 (51.1%) patients.

Asthma was diagnosed by attending physician in 143/401 (35.7%) patients. The asthma clinical stage (intermittent, mild persistent, moderate persistent, severe persistent) is reported in Table 2.

AR was diagnosed by attending physician in 365/401 (91%) patients. The AR clinical stage (mild intermittent, mild persistent, moderate/severe intermittent, moderate/severe persistent) is reported in Table 2.

Atopic dermatitis was diagnosed in 33/401 (8.2%) and conjunctivitis in 238/401 (59.4%) patients.

Two hundred thirty nine patients (59.6%) had received at least one symptomatic treatment in the latest 6 months. The treatments were: anti-histamines in 152/401 (38%), short acting β2 agonists in 45/401 (11.25%), long acting β2 agonists in 45/401 (11.2%), nasal topic steroids in 72/401 (18%), bronchial topic steroids in 69/401 (17.2%) and anti-leukotrienes in 16/401 (4%) patients.

Three hundred twelve patients (77.8%) showed positive results to SPT, while 89 (22.2%) negative results. Table 4 shows all the positive results to the different allergens and their distribution according to the grade of positivity from 1 + to 4 +.

**Table 4:** **Positive results to skin prick tests**

**Allergen**	**Positive result (%)**	**Grading of positive results**			
		**1 + (%)**	**2 + (%)**	**3 + (%)**	**4 + (%)**
Dermatophagoides pteronyssinus	146(36.4%)	22(15.1%)	36(24.7%)	50(34.2%)	38(26%)
Dermatophagoides farinae	142(35.5%)	23(16.2%)	43(30.3%)	41(28.9%)	35(24.6%)
Grass pollen	174(43.6%)	17(9.8%)	47(27%)	59(33.9%)	51(29.3%)
Olive pollen	93(24.2%)	9(9.7%)	25(26.9%)	31(33.3%)	27(29%)
Cat epithelium	82(21.6%)	15(18.3%)	37(45.1%)	21(25.6%)	9(11%)
Parietaria pollen	78(19.5%)	5(6.4%)	16(20.5%)	15(19.2%)	41(52.6%)
Ragweed	69(17.6%)	7(10.2%)	9(13%)	28(40.6%)	25(36.2%)
Mugwort	56(14.5%)	8(14.3%)	24(42.8%)	16(28.6%)	8(14.3%)
Tree pollen (birch, hazelnut,	51(13%)	6(11.8%)	21(41.2%)	11(21.6%)	12(23.5%)
Cypress pollen	49(13.6%)	12(24.5%)	22(44.9%)	10(20.4%)	5(10.2%)
Dog epithelium	48(13%)	17(35.4%)	23(47.9%)	7(14.6%)	1(2.1%)
Alternaria	40(10.3%)	9(22.5%)	18(45%)	11(27.5%)	2(5%)
Cockroach	1(0.7%)	1(100%)	0(0%)	0(0%)	0(0%)

The analysis by the Cohen’s kappa coefficient showed that all questions had a almost perfect concordance between pre and post-test, with value higher than 0.81.

**Figure 1:**
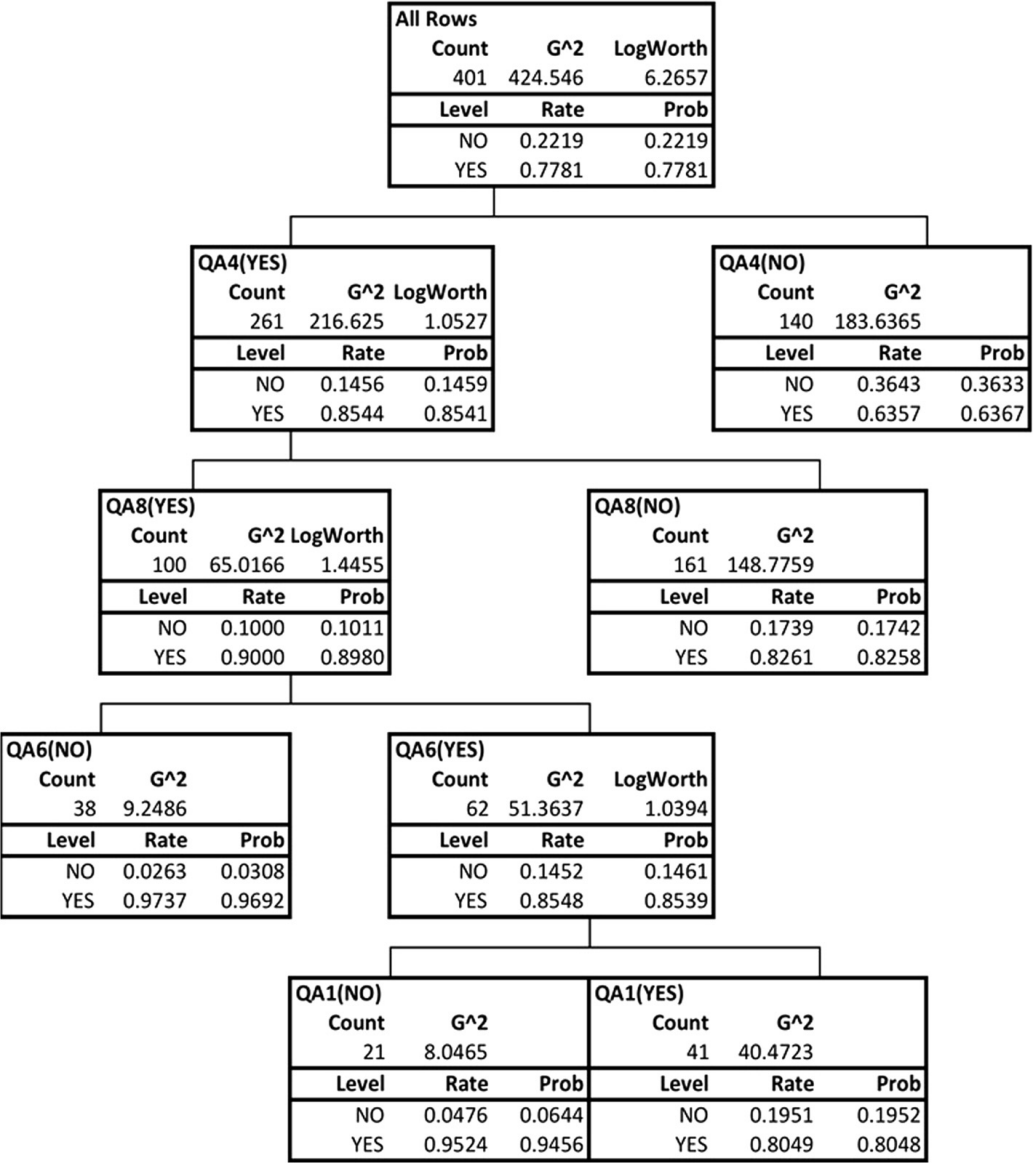
**Decision-tree growth CHAID.** The decision tree based on such correlation the most important questions selected by decision-tree growth CHAID were Q4, Q8, Q6 and Q1.

The analysis of the correlation between the response to each question and the result of SPTs allowed to build a decision tree (Figure 1). Based on such correlation the most important questions selected by decision-tree growth CHAID were Q4, Q8, Q6 and Q1.

Answering yes to the question 4, 6 and 8 gave a probability to have a positive SPT of 85%. Answering yes to Q4, Q6 and Q8 and no to Q1 gave a probability to have a positive SPT of 94.6%. The solidity of the used model was demonstrated by an AUC value corresponding to 0.675, defining a correct classification of the positive answers of around 80%. Thus, the final RAP questionnaire, to be proposed for clinical use, is composed of 4 questions as reported in Table 5.

**Table 5:** **The final 4-question RAP questionnaire**

1	Do you have parents/relatives suffering from rhinitis and/or asthma?
4	Your nasal/ocular complaints do usually start or worsen during the spring?
6	Did you ever had cough or shortness of breath, even during exercise?
8	Do you use nasal sprays frequently?

Y/N answers allowed.

## Discussion

AR is a high prevalence disease. Its impact on the quality of life, also due to the numerous comorbidities (including asthma, sleep disturbances, otitis, rhinosinusitis) is recognized on scientific bases [13-15] and is well known to allergy specialists. However, the general perception of AR is that of a mild disease, because it is not life-threatening, and its economic burden is generally underestimated. Indeed, recent data showed that patients perception is different, the main complaints being the prolonged duration of symptoms, the impairment of sleep and emotional life and the cost of the disease compared with little perceived benefit [5]. Primary care physicians and also pharmacists are the first-line referral for this problem, which is usually underestimated and managed with over-the-counter medications [16]. On the other hand, a correct management can be performed only after AR is properly diagnosed. The etiologic diagnosis of AR is based on the correlation of history data and results of test to detect IgE-mediated sensitization [17], but this diagnosis is rarely done in general practice [18]. Actually, primary care physicians are not familiar with SPTs with allergen extracts and if in vitro IgE tests are requested there is the problem of their interpretation, that often is not simple. This makes useful the availability of easy-to-use instruments to diagnose or, at least, suspect AR in the primary care physician setting. For these reasons we designed this study to set up and validate a self-administered diagnostic questionnaire, the respiratory allergy prediction (RAP) test, that can be self-completed by the patient, the results of which can prompt the physician to ask for a specialty referral. The items of the RAP questionnaire, set up by a panel of experts and preliminary evaluated in a pilot study, addressed the family history of rhinitis and/or asthma, the occurrence of nasal and ocular symptoms during the year, the duration and seasonality of symptoms, the worsening of symptoms in dusty environment, the occurrence of bronchial symptoms and of nocturnal awakenings caused by them, and the frequent use of nasal sprays. The results of SPT were used to confirm the diagnosis of AR, and the statistical analysis assessed the probability of answering yes to the questions to be followed by positive SPT. SPT was chosen because it is suggested in the consensus document Allergic Rhinitis and its Impact on Asthma (ARIA) as the first-line test [2]. The present study shows that the RAP test allows to suspect AR with an high probability to obtain confirmation by positive SPT. In particular, answering yes to the question 4 (“Your nasal/ocular complaints do usually start or worsen during the spring?”) gave a probability of AR of 85.4%. Indeed, the strong power of the question concerning the seasonality of symptoms highlights an actual difference between AR and non AR. In fact, most non AR are perennial, while the occurrence of symptoms during the spring is strictly related to allergic sensitization to pollens. Thus, answering no to question 4 draws the attention on non AR and makes useful further investigations, such as nasal cytology, that enables to identify clinical forms as eosinophilic non-allergic rhinitis (NARES), non-allergic rhinitis with mast cells (NARMA), neutrophilic non-allergic rhinitis (NARNA), and eosinophil-mast cell non-allergic rhinitis (NARESMA) [19]. Based on the number of questions, many combinations of responses (as shown in the Figure 1 on the decision tree) were generated. The highest probability (94.6%) to have positive results to SPTs was associated to the combination of answering yes to Q4, Q6 and Q8 and no to Q1. This may appear surprising considering that Q1 concerns the positive family history for rhinitis and/or asthma, but such finding is simply statistical. For the purpose of the RAP, the probability of 85% given by the combination of answering yes to Q4, Q6 and Q8 meets the aim to identify patients to be evaluated by the allergist.

The strength of this study is to make available a simple tool to be used in primary care to confirm, by its high predictive value, the clinical suspect of respiratory allergy and thus suggesting the real indication to perform allergy testing. The limitation concerns the fact that the allergens used for skin tests were those from a European standard panel, including the 12 most frequently responsible inhalant allergens. This makes possible that patients sensitized to more rarely responsible allergens may not be correctly identified. Also, the age range of 14 to 62 years makes the RAP test not usable in children, who deserve a specific study.

The prevalence of AR in the studied population of subjects with nasal symptoms, as demonstrated by the concordance of symptoms and positive SPT was around 70%. This value is in substantial agreement with recent epidemiological data [20,21]. The rhinitis symptoms are unable to directly suggest the diagnosis, because they are similar in AR and in nonallergic rhinitis, and thus many patients (approximately 30% in our study) with nonallergic rhinitis are referred to allergists to perform diagnostic tests. A number of questionnaires for AR were proposed for epidemiological investigations [22], but all patients are generally also evaluated by allergy tests and no data are available on the ability of the questionnaire to identify the really allergic patients. Thus, the RAP questionnaire is the first aimed at this goal. Currently, the questionnaire may only apply to Italy and a validation in different countries is needed.

The use of RAP test, and of similar questionnaires to predict allergy in patients with rhinitis symptoms, possibly adapted to local environmental natural occurrence of inhalant allergens, has several implications in terms of costs, by reducing the number of subjects to be referred to allergists for testing, as well as of practice, enabling primary care physicians and pharmacists to screen subjects with suspected AR, and of management, indicating the appropriate treatment, that is substantially different in allergic and nonallergic rhinitis, especially considering disease-modifying intervention such as allergen immunotherapy.

In conclusion, the RAP test can be proposed as an useful tool to be used by primary care physicians and specialists other than allergists when evaluating patients with rhinitis. Answering yes to the questions concerning the seasonality of symptoms, to have or have had cough and shortness of breath and to use frequently nasal sprays gives high probability to have respiratory allergy and suggests a real need of allergy testing. This is likely to improve the suitability of patient referral to the allergist and thus to reduce the number of inappropriate tests.

## Abbreviations

PCP: Primary care physicians
RAP: The Respiratory Allergy Prediction
SPT: Skin prick test
AR: Allergic rhinitis
GEIRD: Gene environment interactions in respiratory diseases
SCUAD: Severe chronic upper airways disease
ROC: Receiving operating curve
AUC: Area under the curve
CHAID: Chi-squared automatic interaction detector
NARES: Eosinophilic non-allergic rhinitis
NARMA: Non-allergic rhinitis with mast cells
NARNA: Neutrophilic non-allergic rhinitis
NARESMA: Eosinophil-mast cell non-allergic rhinitis
